# Improving epilepsy monitoring using long-term, in-home-bi-modal seizure monitoring device: clinical utilities and obstacles from a pilot study

**DOI:** 10.3389/fneur.2025.1609838

**Published:** 2025-07-10

**Authors:** Jaeso Cho, Young Jun Ko, Yoon Gi Chung, Anna Cho, Hunmin Kim

**Affiliations:** ^1^Department of Pediatrics, Seoul National University Bundang Hospital, Seongnam, Republic of Korea; ^2^Department of Pediatrics, Chung-Ang University Gwangmyeong Hospital, Gwangmyeong, Republic of Korea; ^3^Department of Pediatrics, Chung-Ang University College of Medicine, Seoul, Republic of Korea; ^4^Department of Pediatrics, Seoul National University College of Medicine, Seoul, Republic of Korea

**Keywords:** epilepsy, seizure monitoring, wearable device, bi-modal signals, clinical utility

## Abstract

**Objective:**

Our study aimed to evaluate a long-term, in-home, bi-modal wearable device for seizure monitoring in epilepsy patients, assessing its applicability, clinical utility and identifying obstacles in real-life settings.

**Methods:**

A prospective pilot study involved 14 epilepsy patients at Seoul National University Bundang Hospital from May 26, 2021, to January 31, 2022. Patients used a wearable device developed for the study, featuring four-channel electroencephalogram and accelerometer sensors. Neurologists provided instructions for device usage, and bi-modal signals were recorded during daily activities. Seizures were annotated through comprehensive data review, and clinical suggestion was provided based on annotated daily seizure frequency, identification of different seizure types, and monitoring seizure patterns throughout the day.

**Results:**

Nine patients (64.3%) used the device for over 100 h, totaling 3,724 h of monitoring and capturing 1,609 seizures. The device successfully recorded various seizure types, including focal, focal with bilateral spread and generalized/bilateral onset which were further annotated by reviewers. Based on the annotated data, we were able to provide clinical suggestions based on number of seizures, identification of ambiguous seizures and monitoring of diurnal seizure pattern. Device discontinuation factors included skin irritation, patients’ unwillingness due to device appearance, and caregivers’ reluctance to use the device.

**Conclusion:**

This study demonstrates the feasibility and clinical utility of a long-term, in-home, bi-modal wearable device for seizure monitoring in epilepsy patients. The long-term data recorded by the device provided valuable clinical insights, facilitating different treatment suggestions. Addressing issues such as device comfort, appearance, and ease of use is essential for enhancing patient and caregiver adherence. The findings support the potential of wearable technology to improve epilepsy management through seizure monitoring in real-life setting.

## 1 Introduction

Seizure is a key symptom of epilepsy and are essential for diagnosis and treatment (1, 2). Accurate seizure monitoring is crucial for effective treatment, allowing for adjustments in antiseizure medication (ASM) dosage and type, and assessing the need for alternative treatments. Early identification of prolonged seizures is particularly important to ensure patient safety and prevent potential harm or risks like SUDEP (Sudden Unexpected Death in Epilepsy) (3).

In real-life situations, confirming and accurately monitoring seizures in everyday lives heavily relies on patient and caregiver reports. However, studies have revealed significant discrepancies between actual seizure frequency and the seizure diaries reported by patients and caregivers (4, 5), with many individuals being unaware of these differences (6). As a result, healthcare professionals and patients/caregivers consistently emphasize the need for objective seizure monitoring. The increasing popularity of smartwatches and the growing trend of mobile health management have led to a rise in research on seizure monitoring using wearable devices, with these devices gradually finding their place in clinical settings (7).

The long-term video electroencephalogram (VEEG) examination conducted in hospital settings is widely regarded as the gold standard for diagnosing seizures. However, it has notable limitations in terms of time, cost, and the controlled environment that differs from patients’ everyday lives (8). In response to these limitations, alternative methods for seizure monitoring, such as subcutaneous and ambulatory electroencephalogram (EEG), have been proposed (9, 10). Nonetheless, patients still encounter challenges when it comes to comfortable long-term non-invasive monitoring, leading to recent research focusing on seizure monitoring using wearable devices to address these drawbacks.

Wearable devices exhibit variations in sensitivity and specificity, which are influenced by the characteristics and modality of seizures, posing challenges in ensuring data quality (11, 12). To tackle this issue, studies have consistently utilized bi-modal devices to enhance seizure monitoring ability and capitalize on the strengths of each modality (13–15). However, there is limited documentation on the long-term real-life application of these devices and scarce research on the experiences of patients and healthcare professionals regarding their extended use (16–18).

In this study, we have developed a seizure monitoring system to monitor seizures using a long-term, in-home, bi-modal wearable device with 4 channel electroencephalogram and accelerometer sensors for seizure monitoring in epilepsy patients. A prospective pilot study was performed on 14 epilepsy patients to evaluate clinical utilities, advantages of multi-modal signals and identified obstacles of in-home wearable seizure monitoring device in real-life setting.

## 2 Methods

### 2.1 Patient selection

From May 26, 2021, to January 31, 2022, we prospectively enrolled 14 patients diagnosed with epilepsy at Seoul National University Bundang Hospital. Inclusion criteria were as follows: patients diagnosed with epilepsy by our neurologists, capable of integrating wearable systems into their daily routines, experiencing seizures or seizure-like events, and patients or their caregivers able to identify seizures. We further selected individuals with a high seizure frequency who demonstrated strong enthusiasm for participation into the pilot study of the device. We further selected individuals with a high seizure frequency who demonstrated strong enthusiasm for participation into the pilot study of the device. Exclusion criteria included patients and families anticipated to have low digital literacy—defined as an inability to proficiently operate digital applications and wearable devices. In addition, any patient who, due to dermatologic issues, was unable to use the wearable system continuously for at least 1 month was also excluded. Withdrawal criteria were defined as inability to wear the wearable systems for an extended period, difficulties in data transmission due to poor dexterity or technical problems, and determination by neurologists during participation that the patient was ineligible for monitoring based on medical reasons.

This study was approved by the Institutional Review Board (IRB) of Seoul National University Bundang Hospital (IRB No. B-2103-673-311). Informed consent was obtained from all the participants. This study was conducted in accordance with the Declaration of Helsinki.

### 2.2 Development of seizure monitoring system using long-term, in-home bi-modal seizure monitoring device

The wearable device was developed by SK Biopharmaceuticals Co., Ltd. (hereinafter referred to as SKBP) specifically for this study. The device comprises sensors and control modules. The sensor module features two disposable hydro-gel type electrodes designed for placement behind the ears at the left and right behind-the-ear positions, denoted by LB and RB, respectively. Upon the need to record EEG from frontal lobe, two additional electrodes may be connected to the sensors positioned behind the left ear, with these electrodes placed on the forehead at the left and right forehead positions, denoted by LF and RF, respectively. The number of electrodes were chosen to maximize the applicability to daily lives while ensuring high sensitivity to various types of seizures. All electrodes are secured to the skin using adhesive stickers, eliminating the need for conductive gel. A three-axis accelerometer was positioned behind the right ear sensor and was connected to the sensor module.

**Figure 1:**
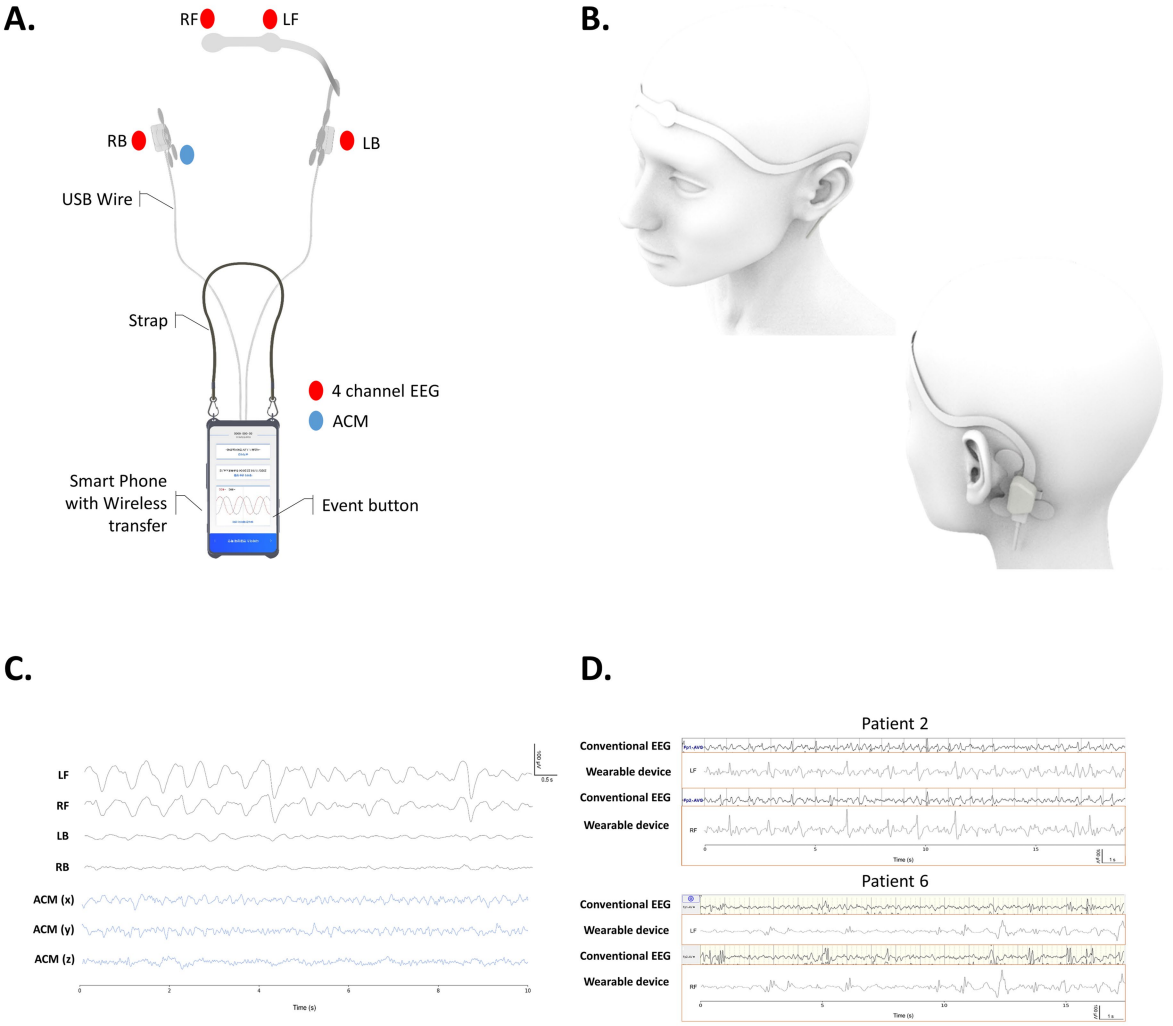
Schematic view of wearable seizure monitoring device used in this study. **(A)** Overview of the system attached to the smart phone system. **(B)** Processed image of the device worn by a patient. **(C)** The example of data recorded from bi-modal wearable device. **(D)** Scalp EEG of corresponding channel of conventional EEG and on bi-modal wearable device compared in patient 2 and patient 6 (Fp1 for LF, Fp2 for RF). The analysis involves a comparison between the EEG recordings of patients conducted in the hospital, rather than simultaneous recordings. LF, left forehead position; RF, right forehead positions; LB, left behind ear position; RB, right behind ear positions; EEG, electroencephalogram; ACM, accelerometer.

The control module, integrated into a Galaxy S10 + Android smartphone (Samsung Electronics, Suwon, Republic of Korea), handles digital signal processing, data upload, and user interaction. It powers the wearable system for up to 12 continuous hours, then requires a 1–2 h recharge. Because data transmission is disabled during charging, recordings obtained in that interval are unavailable for review. The schematic presentation of the device used in our study is shown in Figures 1A,B. Once the sensors and electrodes are in place, patients or caregivers can launch the mobile app. A single tap of the start button initiates recording; tapping it again stops the session, and a subsequent tap immediately resumes recording, enabling quick recovery from any accidental termination. During work hours, users were notified when the device was stopped accidently through telephone call. Seizure events can be logged in real time by pressing the event button during recording. The device was not water-resistant; therefore, users were instructed to pause the recording and remove it before any water exposure, such as showering.

### 2.3 Data acquisition

To ensure the correct usage of the device and to address potential concerns, two neurologists (HK and YK) provided comprehensive instructions for recording bi-modal signals in home settings. All participants were directed to capture EEG, and ACM signals as part of their bi-modal signal recording, utilizing four electrodes positioned behind the ears and on the forehead.

During recording, all the bi-modal signals were uploaded to the server every 5 min. Once the recording was confirmed, bi-modal signals were downloaded from the server. To facilitate further analysis, all the signals were converted to European data format (EDF) files using EDF browser.^1^ The bi-modal signals were recorded at a sampling frequency of 250 Hz.

To enhance signal quality, we applied specific filters to each signal type. EEG signals were bandpass filtered between 0.5 and 70 Hz, and accelerometer (ACM) signals (x, y, z axes) between 0.5 and 30 Hz. A 60 Hz notch filter removed powerline interference. This processing yielded four-channel EEG signals (LB, RB, LF, and RF) and three-channel ACM signals (ACM x, ACM y, ACM z) as bi-modal data. Relative to the standard 10–20 montage, the device recorded EEG from F7 (LF), F8 (RF), T3 (LB), and T4 (RB) in a referential configuration. Data were sampled at 250 Hz with 24-bit resolution. The downloaded data were thoroughly analyzed using MATLAB software (MathWorks, Natick, MA, United States) to ensure accurate and standardized processing for further examination and interpretation. To ensure the signal quality, the device continuously checked electrode contact by monitoring for signals < 62.5 Hz on the reference leads. If such a signal was detected, the application alerted the user with an audible tone and pop-up prompt, instructing them to reposition the electrodes until proper contact was restored.

Throughout the recording period, patients and caregivers were instructed to document the times at which they observed or suspected the occurrence of seizures, utilizing the mobile application provided. We referred to this time as event time. All the reported event times were promptly uploaded to the server as soon as the patients or caregivers pressed the confirm button.

### 2.4 Comparison of EEG quality obtained from the wearable device to conventional EEG

To verify the quality of the EEG recordings obtained from the wearable device, these were evaluated against scalp EEG recordings from the same patient, which were not captured concurrently. Figure 1C displays the processed images from the wearable system, the signals acquired by the device, and a comparison of the EEG quality with that of conventional EEG (Figure 1D). Not all patients go through video EEG monitoring for seizure classifications and conventional 30 min EEGs were chosen as reference EEGs.

### 2.5 Seizure annotation and clinical suggestion

Two neurologists (H. K. and Y. K.) reviewed all bi-modal recordings to verify seizures in the 14 patients. First, we examined and annotated every seizure reported by patients or caregivers. Next, we used these annotated EEG and bi-modal patterns to review the entire dataset for matching signatures, identifying additional electroclinical events. Further efforts were made to identify non-motor seizures based on patient/caregiver’s semiology notes. The ictal period was defined as the interval from seizure onset to termination. Patients who used the device > 100 h were classified as “dedicated” users; those with < 100 h were deemed “discontinued” users.

**Figure 2:**
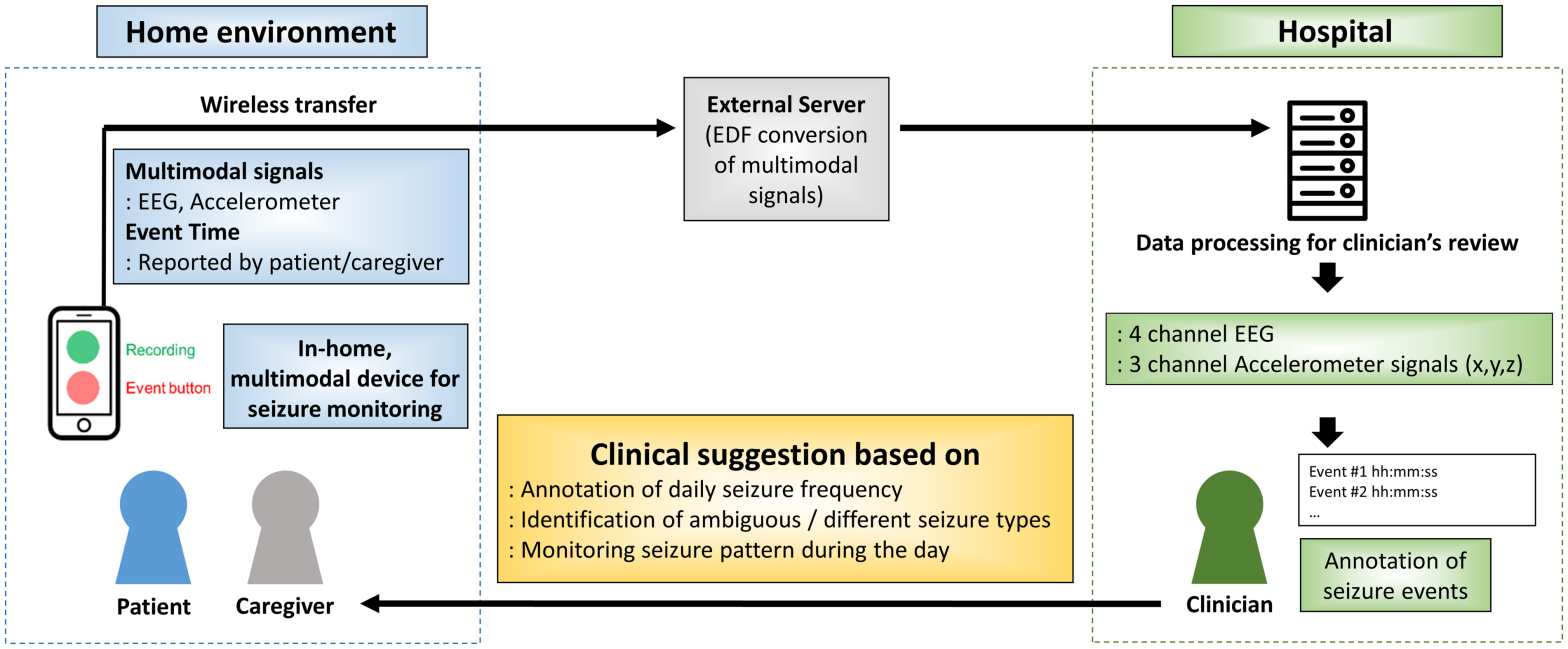
Overall process for data acquisition, seizure annotation and clinical suggestion. EDF: European data format; EEG: electroencephalogram; ACM: accelerometer.

Based on the acquired data, annotated daily seizure frequency, identification of different seizure types and seizure distribution throughout the day was analyzed for each patient. Based on the results, clinical suggestions was provided if indicated. The overall process for data acquisition, seizure annotation and clinical suggestion is shown in Figure 2.

### 2.6 Hourly seizure frequency analysis

The analysis conducted to focus on the hourly seizure rates, defined as the number of seizures occurring per hour, among patients who engaged in bi-modal signal recording for over a month and experienced at least 10 seizures verified by neurologists. Hourly seizure frequency was annotated to assist patients and caregivers in identifying seizure hotspots during the day, thereby allowing them to recognize periods when they should be more attentive to clinical symptoms.

## 3 Results

### 3.1 Patient characteristics and seizure annotations in dedicated users

Fourteen patients (7 male, 7 female) were enrolled. Median age at study entry was 17 years (range 7–28), and median epilepsy onset was 5.5 years (0.4–16). Twelve had focal epilepsy and two had Lennox–Gastaut syndrome; etiology was structural in eight and unknown in six. EEG showed focal epileptiform discharges in 10 patients, multifocal discharges in two, and generalized discharges in two. The clinical characteristics of patients participated in this study is summarized in Table 1.

**Table 1:** Demographic and clinical characteristics of enrolled patients.

	Sex	Age (yr)	Onset age (yr)	Epilepsy diagnosis	IED	MRI	Etiology	Seizure frequency	Daily activity	Number of ASMs
1	F	20	5	LRE	Focal	NS	Unknown	Several/W	Ambulatory, ID	4
2	M	23	0.4	LGS	Generalized (Bifrontal)	Mild atrophy	Unknown	Frequent/D	Bed-ridden	5
3	F	22	1	LRE	Focal	NS	Unknown	Frequent/D	Bed-ridden	4
4	M	10	4	LRE	Focal (Multifocal)	HIE	Structural	Frequent/D	Bed-ridden	5
5	F	10	7	LRE	Focal	NS	Unknown	Frequent/D	Ambulatory	3
6	F	9	2	LRE	Focal (Multifocal)	Band heterotopia	Structural	Frequent/D	Ambulatory, ID	4
7	M	18	6	LRE	Focal	Infarction	Structural	Several/W	Ambulatory	1
8	F	12	9	LRE	Focal	Mild atrophy	Structural	Frequent/D	Ambulatory, ID	4
9	M	13	11	LRE	Focal	PVL	Structural	Several/W	Ambulatory, ID	2
10	M	7	0.5	LRE	Focal	NS	Unknown	Several/W	Ambulatory	2
11	F	18	15	LRE	Focal	ACC	Structural	Several/M	Ambulatory, ID	3
12	M	16	7	LRE	Focal	Tumor (DNET)	Structural	Several/M	Ambulatory	2
13	F	28	16	LRE	Focal	HS	Structural	Several/M	Ambulatory	0
14	M	23	7	LGS	Generalized (Bifrontal, GSW, GPFA)	NS	Unknown	Frequent/D	Ambulatory, ID	5

IED, interictal epileptiform discharge; MRI, magnetic resonance imaging; ASM, antiseizure medication; LRE localization-related epilepsy; LGS, Lennox–Gastaut syndrome; GSW, generalized spike–wave complex; GPFA, generalized paroxysmal fast activity; NS, not significant; HIE, hypoxic Ischemic encephalopathy; PVL, periventricular leukomalacia; ACC, agenesis of corpus callosum; DNET, dysembryoplastic neuroepithelial tumor; HS, hippocampal sclerosis; W, week; D, day; M, month; ID, intellectual disability.

**Figure 3:**
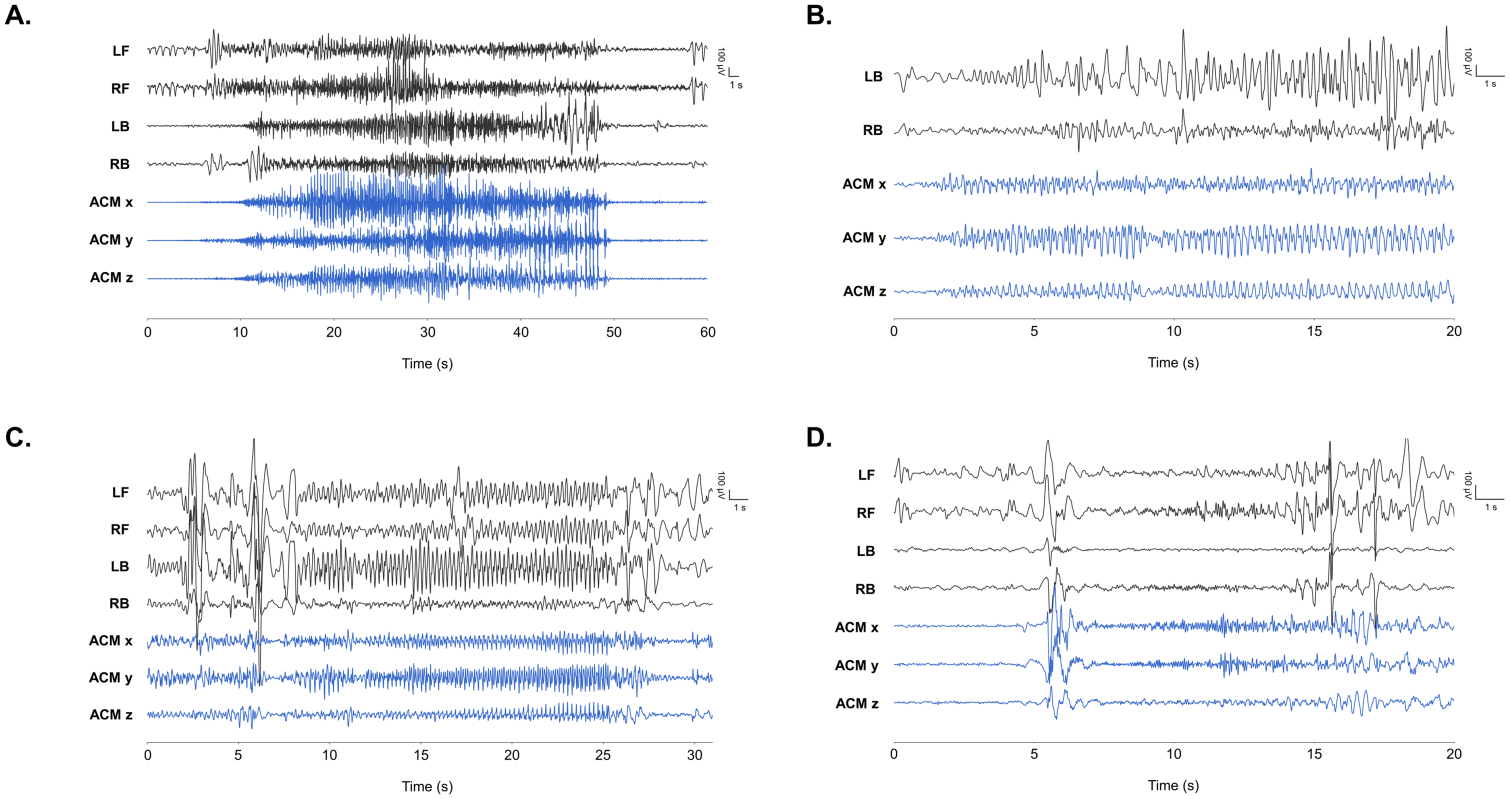
Representative EEG findings during seizure events obtained by the wearable device in our study. LB/RB and LF/RF refers to EEG positioned at left and right behind ears, and left and front, respectively. ACM x/y/z refers to x/y/z axes of the accelerometer positioned at right behind ear. Time frame of each finding is noted on the x-axis of each graph in seconds. **(A)** Tonic seizure annotated in patient 2. **(B)** Focal onset seizure with chest tightness in patient 7. **(C)** Hypomotor seizure with lip cyanosis in patient 8. EEG findings show rhythmic 3.5–4 Hz high voltage spike wave discharges originating from multiple areas. Ictal discharges with shorter time frame is depicted in red dotted box. **(D)** Myoclonic seizure with generalized discharge in patient 14.

Nine of the 14 patients (64.3%) met the “dedicated user” criterion (> 100 h of use). In this group, 1,609 seizures were recorded over 3,724 h, encompassing focal onset, focal-to-bilateral tonic–clonic, tonic, and myoclonic events (Figure 3). These annotated seizures enabled clinical suggestions (Table 2).

**Table 2:** Summary of clinical outcome of epilepsy patients after using wearable seizure monitoring device.

Patient #	# of reported seizures by patient/caregiver	# of annotated seizure events	Monitoring device usage time (hours)	Clinical suggestion	Complaints during device use
Dedicated users					
2	61	43 (24 Generalized, 19 Focal)	757.9	Annotation of daily seizure frequency → clinical suggestion to caregiver	Skin irritations
3	53	5 (5 Focal)	233.9	Annotation of daily seizure frequency → clinical suggestion to caregiver	Skin irritations, caregiver’s reluctance
6	25	13 (13 Focal)	100.4	Annotation of daily seizure frequency → clinical suggestion to caregiver	Skin irritations
7	24	19 (19 Focal)	108.8	Identification of ambiguous seizure types → ASM adjustment led to seizure free state	
8	135	1,422 (1,422 Focal)	957.7	Annotation of daily seizure frequency → clinical suggestion to caregiver	
10	7	0	110.2	Ambiguous symptom monitoring → clinical suggestion of non-epileptic events	Caregiver’s reluctance
12	11	4 (4 Focal)	403.0	Identification of ambiguous seizure types → ASM adjustment led to seizure free state	
13	7	0	507.3	Ambiguous symptom monitoring → clinical suggestion of non-epileptic events	
14	287	103 (89 Generalized, 14 Focal)	414.9	Identification of ambiguous seizure types → ASM adjustment for better seizure control	
Discontinued users					
1	0	0	10.4		Patient’s unwillingness
4	13	0	3.4		Skin irritations, caregiver’s reluctance
5	4	0	5.6		Patient’s unwillingness
9	7	0	64.0		Patient’s unwillingness
11	5	0	46.6		Patient’s unwillingness

### 3.2 Clinical suggestion based on seizure monitoring

#### 3.2.1 Annotation of daily seizure frequency

Patient 8, a 12-year-old girl recovering from FIRES (Febrile Infection Related Epilepsy Syndrome), had daily intractable convulsive seizures. Over 116 days she used the device for 958 h, which captured 1,422 seizures. Her mother’s diary integrated into the app, typically recorded fewer than 10 clinical events, describing motion arrest and head version that evolved into bilateral tonic–clonic seizures (Figure 3C). The marked discrepancy between device-annotated seizures, marked 36.5 seizures/day, and caregiver-reported counts prompted for clinical suggestions and subsequent ASM adjustments.

#### 3.2.2 Identification of ambiguous/different seizure types and utilization of multi-modal signals

Patient 7 is an 18-year-old male diagnosed with lesional focal epilepsy. He is a survivor of Langerhans cell histyocytosis (LCH) and has encephalomalacia in both occipital lobes as a result of hemorrhagic infarction experienced during chemotherapy. The patient frequently experienced episodes of chest tightness followed by decreased consciousness. Over 17 days, he used the device for 109 h. Suspecting the ictal focus was in his brain lesion, we employed a two-channel EEG positioned behind the ear. During these events, ictal EEG changes from the behind-the-ear channels confirmed a focal seizure originating from the encephalomalacia. The recorded data displayed repetitive spike or spike–wave discharges indicative of a seizure (Figure 3B). After confirming an electroclinical seizure, we increased the oxcarbazepine dosage. Following clinical suggestion based on identification of ambiguous seizures, he is now free of seizures with chest tightness.

Patient 14 is 23-year-old male with Lennox–Gastaut syndrome. He is ambulatory but he has severe intellectual disability. As the patient was noted to have multiple seizure types, multi-modal signals and parents’s reports were analyzed together to identify different seizure types. Over a period of 52 days, the device was used for a total of 415 h. Our analysis enabled us to identify 103 seizures, which encompassed various seizure types. Using the caregiver’s descriptions of semiology, we identified three seizure types based on EEG and accelerometer signals. Focal onset hypomotor and tonic seizures were recognized through evolving spike–wave discharges in EEG readings and caregiver annotations. Myoclonic seizures were detected via abrupt changes in accelerometer data accompanied by EEG alterations. Clinical suggestion was provided to the family that the observed motion arrests and myoclonic movements were indeed seizures. After adjusting ASMs, the number of annoted seizures were decreased to 7.8 times/day to 2.5 times a day. Representative EEG findings are shown in Supplementary Figure S1.

Patient 12 is a 16-year-old male who underwent partial surgical removal of a dysembryoplastic neuroepithelial tumor (DNET) in the right temporal uncus. After surgery, his habitual seizures ceased, but he began experiencing episodes of chest pain lasting 20–30 s once or twice a month. Cardiology evaluations failed to identify a cause. Enrolled in our study, we detected spike discharges on EEG correlating with his chest pain episodes, confirming them as epileptic seizures. After adjusting his antiepileptic medications, the patient has been seizure-free for 1 year.

#### 3.2.3 Monitoring seizure pattern throughout the day

We categorized patients’ hourly seizure data to analyze seizure frequency by hour. Despite intermittent device usage and high seizure counts, each patient’s hourly seizure distribution showed distinct patterns. Patient 2 had frequent seizures primarily during sleep. Patient 4 experienced frequent seizures in the morning and afternoon. Patient 8 had fewer seizures during sleep, while patient 14 consistently had seizures throughout the day except during sleep (Supplementary Figure S2). Although ASMs were not adjusted based on these seizure-prone periods, families received clinical suggestions to increase awareness for enhanced patient safety.

### 3.3 Factors leading to discontinuation or undesirability of wearable device

During the course of the study, a total of 5 patients (35.7%) discontinued the use of wearable devices due to various external factors. Within this group, one patient stopped using the device owing to skin irritations and the caregiver’s reluctance to use the device. Four other patients discontinued use due to their own unwillingness to use the device (Table 2).

#### 3.3.1 Skin irritation

Skin irritation was the primary side effect, occurring only in patients who used the wearable device for over a month (patients 2, 3, 4, and 6). No skin irritation was reported with short-term use. Patient 4 discontinued the device after redness developed, leading to spontaneous improvement. In patient 6, although the device successfully monitored seizure activity from the frontal lobe, continued use was challenging due to discomfort from electrode-induced skin irritation. To address this, only forehead electrodes were used, eliminating the behind-ear electrodes causing irritation. In patients 2 and 4, intermittent use led to spontaneous improvement of skin irritations.

#### 3.3.2 Patients’ unwillingness

Four participants (patients 1, 5, 9, 11) stopped using the device for personal reasons. Each had mild intellectual disability but functioned independently. They found the visible hardware intrusive at school or work, viewing it as a conspicuous marker of their condition; one described feeling “embarrassed” by peers’ questions. This discomfort limited wear time and led to discontinuation.

#### 3.3.3 Caregivers’ reluctance

Caregivers of three bedridden patients found the device and control module burdensome. The intensive care these patients required left little time for electrode application and seizure logging. Consequently, patient 4 discontinued use, whereas patients 3 and 10 persisted and continued to receive clinical suggestions.

## 4 Discussion

### 4.1 Enabling effective long-term, in-home seizure monitoring

For effective seizure monitoring using wearable devices, it is essential to ensure comfortable usage by patients in their daily lives and maintain good signal quality. Previous studies have primarily focused on validating results by combining wearable devices with VEEG in a limited controlled hospital environment (13–16, 19). Few studies, including ours, have investigated the extended use of wearable devices for seizure monitoring in everyday home settings. Previous research, while benefiting from VEEG validation, was limited to controlled hospital environments and may not represent data captured during patients’ daily activities. Additionally, these studies faced cost and time constraints, with examination periods typically confined to a maximum of 1 week. Home-based seizure monitoring studies have primarily reported on wrist-worn devices capturing accelerometer data alone or alongside electrodermal activity (20–22).

In this study, we successfully demonstrated that the majority of the patients comfortably used wearable devices with behind-the-ear EEG during extended daily activities, experiencing minimal discomfort or serious adverse events. Utilizing a smartphone as a control module and battery charger allowed for 12 continuous hours of operation, capturing daily activities without battery constraints. By placing up to four electrodes on the forehead and behind the ears based on each patient’s epileptic focus, we were able to perform seizure monitoring with fewer channels (23–26). The implementation of a three-axis accelerometer aided in characterizing different seizure types during annotations. Based on long-term seizure monitoring, clinical suggestions provided to the patients and families further motivated them to use the monitoring device.

### 4.2 Suggesting clinical utility of long-term seizure monitoring from a pilot study

We demonstrated the possible clinical utility of using wearable devices for seizure monitoring in different epilepsy patients. In clinical practice, accurately assessing seizure frequency is crucial for evaluating and adjusting ASMs. However, previous studies have reported significant discrepancies between patient or caregiver reports and the actual seizure frequency (4, 5, 27). Our study results showed that seizure monitoring using wearable devices can effectively monitor seizures and provide annotation of undetected seizure frequency.

The use of wearable devices provided direct confirmation of ictal EEG changes in patients where the presence of seizures was uncertain. Previous studies have also demonstrated that ambulatory EEG can assist in identifying interictal epileptiform discharges and differentiating seizures in real-life settings of patients (28). However, the inconvenience associated with traditional examination equipment has made it challenging to conduct tests that span several days (8). Considering these findings, we believe that wearable devices can offer patients a comfortable and user-friendly solution for accurate seizure monitoring in their everyday lives.

Using a bi-modal device showed advantages in monitoring various seizure types. We successfully identified myoclonic, tonic, and focal onset seizures with or without impaired awareness. These findings align with prior studies that highlight the superiority of bi-modal wearable devices over single-modality devices, enhancing seizure monitoring rates and effectively identifying diverse forms of seizures (13–15, 29). Although wearable devices emphasizing convenience may exhibit slightly lower sensitivity and specificity in seizure monitoring, the use of bi-modal devices may compensates by increasing the overall monitoring rate. An additional strength of the long-term wearable device was its ability to collect data over extended periods, enabling us to analyze patient-specific seizure patterns at different times throughout the day. Previous studies have reported variations in seizure patterns depending on the time periods and seizure types (8–10). Patients can experience multiple seizure types, and long-term wearable data collection allows for analysis of time-dependent seizure patterns. Some studies suggest seasonal differences in seizures (12, 15). By collecting long-term data, we can examine individual seizure patterns at a more granular level, potentially leading to personalized treatment approaches, including seizure forecasting.

### 4.3 Discontinuation of wearable device

Among the 14 participants, 9 patients (64.4%) maintained their regular routines and collected bi-modal data—including EEG and accelerometer measurements—for at least 100 h (median 403 h) over more than a month. Five patients discontinued device use; only one stopped due to a direct side effect, specifically skin irritation. Skin rash is a documented potential side effect in wearable ECG and EEG research (17). Skin irritation and rash were the most common side effects observed, consistent with previous wearable EEG studies. In all four patients reporting skin rashes, the irritation spontaneously resolved after electrode removal, indicating it was mild and self-limiting. Only one patient discontinued device use, while the other three remained dedicated. Considering the long-term use of wearable devices, developing electrodes that minimize skin irritation would be advantageous for patients relying on these devices over extended periods.

Studies on wearable device usage among epilepsy patients have suggested that the design and appearance of the devices are less significant for adults than previously anticipated (30). The impact of device design and appearance on usage in pediatric populations remains uncertain. In our study, four adolescents discontinued using wearable devices due to unwillingness to wear exposed, visible designs. Future development of wearable devices for seamless integration into daily activities outside the home should give greater consideration to their external appearance.

Three caregivers struggled to find time to apply the device. Since patients often have disabilities, a simpler and more convenient application process is needed. Strong encouragement from clinicians—emphasizing that using the device could improve the child’s disease management—is essential. Special considerations and improvements are necessary to ensure patients and caregivers can actively and effectively use the wearable device (17, 30–32).

In conclusion, we evaluated the applicability of a long-term, in-home, bi-modal wearable device for seizure monitoring in patients with epilepsy. Overall, the wearable device showed promise in providing sufficient seizure monitoring and valuable information for epilepsy management. The device’s ability to capture bi-modal signals, including EEG and ACM signals, enhances its utility in seizure monitoring. Further research is warranted to explore the long-term real-life application of wearable devices and gather more insights from patients and healthcare professionals regarding their experiences and challenges.

### 4.4 Limitations

This study has several limitations. First, seizure detection was confined to electrode sites; identifying extra-coverage events would require additional, region-specific leads. Second, poor connectivity and frequent data uploads accelerated battery drain, underscoring the need for on-device storage and analysis. Third, the system operated for up to 12 h on a single charge; extending runtime is essential for multi-day monitoring. Fourth, because not all patients underwent video-EEG monitoring for seizure classification and no simultaneous conventional EEG recordings were obtained, our ability to validate the wearable device’s detected signals is limited—despite efforts to match background activity and interictal discharges from conventional EEG recordings taken at different time points. Fifth, due to lack of various seizure types detected by our monitoring device, we were unable to stratify and analyze various seizure types and their characteristics. Finally, the absence of an ECG channel restricted multimodal assessment, highlighting the need for a skin-friendly ECG module in future designs.

It should be emphasized that this device is not a diagnostic tool and cannot perfectly classify seizure types. Its utility is greatest in patients whose seizure patterns have already been well-characterized through prior EMU admissions or prolonged video EEG monitoring, as it allows for quantification of seizure burden in the home environment. However, its sensitivity and specificity may be lower for non-convulsive seizures and patients with less seizure frequency compared to controlled inpatient environments. Therefore, while the device can provide valuable Supplementary material, it should not be used as the sole basis for diagnosis or treatment decisions.

## Data Availability

The raw data supporting the conclusions of this article will be made available by the authors, without undue reservation.

## Footnotes

^1^ https://www.teuniz.net/edfbrowser
