# Supplementary material for: Improving epilepsy monitoring using long-term, in-home-bi-modal seizure monitoring device: clinical utilities and obstacles from a pilot study
Source: Front Neurol. 2025 Jul 10;16:1609838. doi: 10.3389/fneur.2025.1609838 (PMC12290470; doi:10.3389/fneur.2025.1609838)
Supplement: Supplementary file 1 [file Supplementary_file_1.docx]

Supplementary Material

# Supplementary Figures


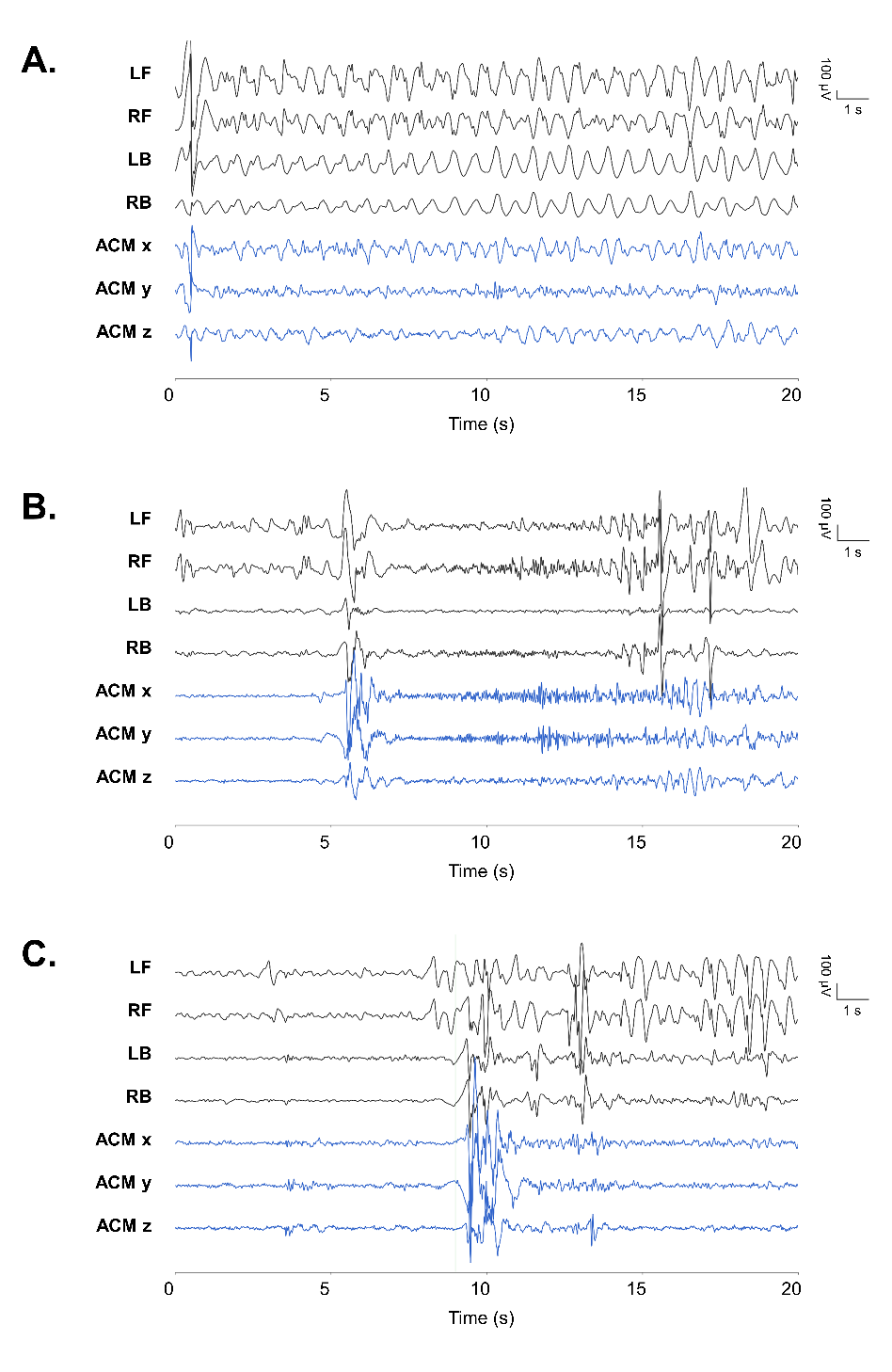


**Figure S1.** Various seizures and corresponding bi-modal recordings in patient 14. A. Focal onset hypomotor seizures. B, C. Depiction of a myoclonic seizure characterized by generalized spike-wave discharges, accompanied by abrupt alterations in accelerometer signals, indicative of a transient change in motion.


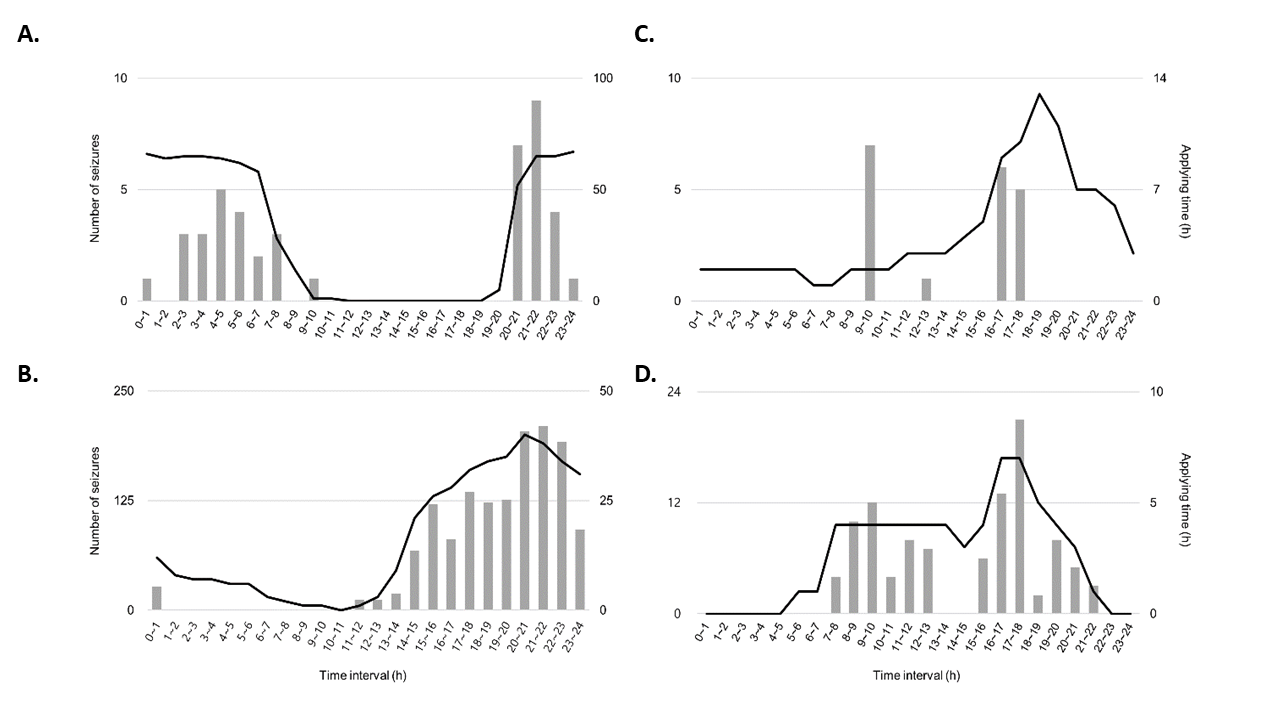


**Figure S2.** Seizure frequency according to time interval of seizure occurrence during wearable device in patient 2 (A), patient 7 (B), patient 8 (C), and patient 14 (D). Gray-colored bars indicate the number of seizures per day and a black-colored line indicates applying time in each time interval.
